# Frost Tolerance Increases With Plant Height Among Co‐Occurring Alpine Species in the Central Tibetan Plateau

**DOI:** 10.1002/ece3.73512

**Published:** 2026-04-29

**Authors:** Ji Suonan, Xuwei Lu, Richard Michalet, Pierre Liancourt, Aimée T. Classen, Wenying Wang, Huichun Xie

**Affiliations:** ^1^ College of Life Sciences Qinghai Normal University Xining China; ^2^ Key Laboratory of Biodiversity Formation Mechanism and Comprehensive Utilization of the Qinghai‐Tibet Plateau in Qinghai Province Xining China; ^3^ UMR EPOC, Université of Bordeaux Pessac France; ^4^ Botany Department State Museum of Natural History Stuttgart Stuttgart Germany; ^5^ Department of Ecology and Evolutionary Biology University of Michigan Ann Arbor Michigan USA

**Keywords:** alpine plant, frost‐tolerance, functional trait, height, interspecific variation

## Abstract

Frost is a major stressor for alpine plants, and a species' ability to resist it can shape their distribution and diversity. While many studies explore how frost tolerance varies with elevation, research on how co‐occurring species differ in their resistance strategies remains limited. We selected twenty‐two co‐occurring plant species from a single alpine community on the Tibetan Plateau and quantified their frost tolerance (LT_50_: the lethal temperature at which 50% of tissue died). We also measured six common functional traits (height, specific leaf area (SLA), leaf dry matter content (LDMC), leaf nitrogen content (LNC), leaf phosphorus content (LPC), and the ratio of leaf nitrogen and phosphorus content (NP ratio)) of all species. Overall, we found there was a lot of interspecific variation in the LT_50_ of alpine plants within the same community. As expected, plant height was the most significant trait associated with LT_50_. However, unlike patterns observed along elevation gradients, in our study taller plants were more frost tolerant than shorter plants. Overall, short stature together with densely packed small leaves of alpine plants may explain why these species are aerodynamically decoupled from the cold air temperature (avoidance strategy) and do not have to rely as strongly on physiological tolerance to frost (tolerance strategy). Multiple strategies by different species in a single community could facilitate species co‐existence under harsh conditions like the Tibetan Plateau. These results can help us to better understand the high diversity of alpine plant communities.

## Graphical abstract

Frost tolerance (LT50) varies greatly among species coexisting in an alpine community. Taller plants are more frost‐tolerant, reversing the expected intra‐community pattern. Evidence for a frost survival trade‐off: avoidance in short plants vs. tolerance in tall plants. Coexistence of multiple strategies may explain high alpine plant diversity.

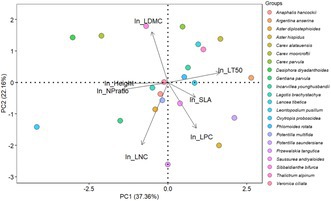

## 1 Introduction

Low temperatures are an important environmental constraint that limits plant species distribution and productivity (Araújo et al. 2013; Sakai and Larcher 1987; Taschler and Neuner 2004). In fact in high‐elevation and high‐latitude ecosystems where frost events are common, low temperature is the primary environmental filter (Inouye 2008; Klein et al. 2018; Körner 2021; Liu et al. 2018; Pescador et al. 2016; Ren et al. 2021; Piao et al. 2019). Frost‐tolerance of plants, expressed as the lethal temperature at which 50% of tissue die (LT_50_), is a commonly measured trait that allows the estimation and prediction of frost damage in plants (Körner 2021; Sakai and Larcher 1987). Plant frost‐tolerance increases along elevation gradients (Bucher et al. 2019; Bucher and Rosbakh 2021; Gast et al. 2020; Sakai and Larcher 1987; Taschler and Neuner 2004). However, how frost‐tolerance differs across species within the same community remains unclear. Since environmental conditions are more consistent within than across communities, trait differences between locally co‐occurring species may be smaller. For instance, plant species from an alpine meadow could have undergone the habitat filtering of low temperatures (Körner 2021), thus, frost‐tolerance may be overall high. However, a recent study conducted along an elevation gradient in Colorado illustrated that co‐occurring species at each elevation displayed a wide range of energy balance trait values (i.e., stomatal conductance, absorptance, and canopy exposure), suggesting that species sorting by strong environmental filtering does preclude the co‐occurrence of species with contrasting strategies (Blonder et al. 2020). Revealing the frost‐tolerance variation of co‐occurring species within a community could provide a theoretical basis for community assembly and biodiversity research in temperature limited ecosystems.

Plant functional traits can capture long‐term adaptations of plants to their environment, thus, traits can define plant ecological strategies (Díaz et al. 2016; Michalet et al. 2023; Reich 2014; Wright et al. 2004). Along an elevational gradient, the height of non‐woody plants has been shown to be positively associated with LT_50_ (since LT_50_ is mostly subzero temperature, lower value indicates higher frost‐tolerance), which supports the general trade‐off in plants between the stress tolerance strategies, and competitive ability (Bucher and Rosbakh 2021; Grime 2006). Generally, freezing resistance involves two contrasting strategies—freezing avoidance by transient supercooling or freezing tolerance by the formation of ice crystals in extracellular spaces (Körner 2021; Larcher 2003; Levitt 1980; Suonan et al. 2024). Previous studies showed that taller plants avoid freezing by supercooling whereas plants near the ground tolerate ice formation (e.g., Sklenář et al. 2010; Squeo et al. 1991). While there is a strong association between plant height and mean annual temperature at global scale (Moles et al. 2014), the extent of aerodynamic decoupling between leaf or canopy temperature and ambient temperature exists in plants along elevation gradient in alpine region. It turns out that plant height is key in determining the extent of the observed temperature decoupling (Liancourt et al. 2020). Short species from high and cold elevations show greater leaf temperature than ambient temperature, whereas taller species from the lower and warmer elevations are cooler than ambient (Liancourt et al. 2020; see also Körner 2021; Michaletz et al. 2016). This phenomenon can result in plant dwarfism in alpine environments, where species exhibiting a prostrate growth form and are aerodynamically decoupled from the cold air temperature (Gates 1980; Grace 1987; Körner 2021). Yet it is still uncertain if plant height plays a role in frost tolerance or frost resistance strategies within a small environmental range, such as a plant community.

Multiple leaf traits on the plant functional economic spectrum are associated with freezing tolerance along elevational gradients (Bucher et al. 2019; Bucher and Rosbakh 2021; Gast et al. 2020). For example, slow‐growing species with low specific leaf area (SLA) and leaf nitrogen content (LNC) values exhibit low rates of cell expansion, lead to smaller and denser leaves, and are generally found in the colder part of a temperature gradient (Körner and Woodward 1987; Poorter and De Jong 1999). Leaf dry matter content (LDMC) can influence the ability of the leaf to store or exchange heat with the environment (Michaletz et al. 2015, 2016). Therefore, a positive association of LT_50_ with SLA and LNC, and a negative association with LDMC may indicate a trade‐off between plant growth and frost tolerance (Bucher et al. 2019; Bucher and Rosbakh 2021; Gast et al. 2020). Additionally, leaf phosphorus content (LPC) and the ratio of LNC and LPC (NP ratio) are generally known to be related to stress resistance (Aerts and Chapin III 1999; Güsewell 2004; Morales et al. 2023), with LPC increasing and NP ratio decreasing along elevation gradients (Chondol et al. 2023; Dvorský et al. 2015). The relationship between traits along long environmental gradients is well studied (Chondol et al. 2023; Dolezal et al. 2019; Dvorský et al. 2015); however, while gradient analyses can identify large‐scale trait variation, the gradient approach may obscure local interspecific plant variation that is essential for understanding species co‐existence.

The Tibetan Plateau is ideally suited for examining plant frost‐tolerance and their relationship with functional traits since it is the highest plateau of the world, and plants are highly exposed to cold events (Miehe et al. 2019; United Nations Environment Programme 2022). Moreover, the highly continental climate on the Tibetan Plateau increases the probability and intensity of frost events (Li et al. 2021; Michalet et al. 2021), yet few quantitative studies assessed frost‐tolerance of alpine plants on the Tibetan Plateau. We selected twenty‐two plant species in an alpine community located at 4500 m elevation on the Tibetan Plateau and simultaneously measured LT_50_ and six functional traits (height, SLA, LDMC, LNC, LPC, and NP ratio) during three years (2021–2023). Our hypotheses are (1) the diversity in frost‐resistance strategy among co‐occurring species should be reflected in a large range of frost‐tolerance (LT_50_); (2) if consistent with the studies along elevation gradients, plant height should be positively associated with LT_50_; (3) leaf traits should correlate with LT_50_. Specifically, we expect a positive correlation with rapid growth or exploitive strategy related traits such as SLA and LNC, and a negative correlation with stress‐tolerance or conservative strategy related traits such as LDMC because the investment in frost‐tolerance occurs at the expense of rapid growth and should be consistent with conservative slow growth.

## 2 Materials and Methods

### 2.1 Study Site and Species Selection

The fieldwork was conducted in the Nagqu Alpine Grassland Ecosystem National Field Scientific Observation and Research Station (hereafter, Nagqu station) located at Kema village on the Tibetan Plateau (31°16′ N, 92°06′ E, 4453 m a.s.l). The study site is in an alpine meadow habitat that experiences a highly continental climate dominated by a southeastern monsoon from May to September and by high pressure from westerly winds in the winter due to a strong rain shadow effects (Li et al. 2021; Michalet et al. 2021). Summers are short and cool, and winters are long and extremely cold. The mean annual temperature is −1.2°C, and the mean annual precipitation is 430 mm, of which over 80% falls during the summer monsoon season (Miehe et al. 2019). Based on temperature data from a weather station at the study site, freezing temperatures (−10°C to 0°C) during growing season are common in this habitat (Figure S1). Based on the prevalence of abundance and biomass, twenty‐two common alpine plant species (three sedges, one legume, and 18 forbs) that are common and co‐occurring in the alpine meadow habitat were selected in this study, including 20 perennial herbs, one annual herb (*Gentiana parvula*) and one cushion shrub (*Dasiphora dryadanthoides*) (Table S1).

### 2.2 Frost Tolerance Measurements

#### 2.2.1 Plant Collection

In each year from 2021 to 2023, from late July to early August, five individuals of each species were randomly selected from natural grasslands between 4450 and 4550 m. We harvested the entire aboveground part of each species, that was immediately placed inside a plastic bag with a damp paper towel and then in a cooler. Within 2 h of collection, all plant samples were transferred to the Nagqu station. After washing the surface of the plants with distilled water, they were placed in a laboratory refrigerator and stored at 15°C before preparation for an experimental temperature treatment. The treatments were applied within 12 h of field collection. We collected fresh plant each day prior to each experimental treatment to control for plant senescence that may occur after collection from the field. For some small species (e.g., *Gentiana parvula, Thalictrum alpinum, Leontopodium pusillum, and Veronica ciliata*), we collected 1–5 extra individuals since the leaves of one individual were not enough for one sample for assessment of frost damage. For the shrub *Dasiphora dryadanthoides*, we collected five twigs instead of the entire aboveground part.

#### 2.2.2 Frost Experiment

We used a programmable freezing chamber (MSX‐2F; Chinese Academy of Agricultural Sciences, Beijing, China) to expose plant samples to seven independent temperature treatments: 0, −2.5, −5, −7.5, −10, −12.5, −15°C. The freezing chamber, which had a window for light, allowed us to have the precise temperature. Our selection of the temperature treatments from 0°C to −10°C encompasses the range of night‐time freezing temperatures that occur at our study site in Nagqu station during the growing season (Figure S1). Whereas −12.5°C and −15°C were meant to represent an extreme condition that lies just beyond what is observed in our ecosystem. We completed the freezing process in three steps. First, plant samples were placed inside the chamber, and the temperature ramped down from 15°C ± 2°C (room temperature at Nagqu station) to the respective target temperature determined by the experimental treatment (0, −2.5, −5, −7.5, −10, −12.5, −15°C; cooling rate is about −4 k/h). Next, we held that temperature constant for 1 h. Finally, the temperature was brought back up to 15°C (about 10 k/h). The extreme frost event in this region usually starts at 12 p.m.–1 a.m. and the lowest temperature occurs at 6–8 a.m. the next day. The cooling treatment in this experiment simulated this process. The specific cooling curves of the different target temperatures and the actual temperature reached are shown in Figure S2. Because we only have one freezer chamber, we conducted all twenty‐two species simultaneously in one chamber, and the seven freezing treatments on seven different days.

#### 2.2.3 Assessment of Frost Damage

Once each temperature treatment was complete, individual plant specimens were removed from the freezing chamber. Leaves of each of five individual plant sample were cut into 2–3 parts. 0.5 g of them were put in test tubes and completely submerged in 10 mL distilled water. The freezing damage was estimated by the conductivity method (Prášil and Zámečník 1998), measuring conductivity of the supernatant, as the conductivity of the samples is proportional to the electrolyte leakage from damaged cells. After 25°C water bath for 7 h, we measured conductivity (*T*_1_) with a conductivity meter (DDS‐307A; Shanghai Leici, Shanghai, China). Then, the tubes were boiled for 1 h to destroy cell membranes (i.e., 100% cells dead), cooled down to room temperature and we again measured the conductivity (*T*_2_). The relative conductivity was calculated as:

(1) $$\text{The relative conductivity}=\frac{T_{1}}{T_{2}}*100\%$$

### 2.3 Functional Trait Measurements

To test relationships between functional traits and frost tolerance, we collected data on six functional traits. They included plant height (the maximum shoot height from the ground, cm), SLA (the ratio of the leaf fresh area to its leaf mass, cm^2^/g), LDMC (the ratio between dry mass and saturated fresh mass, mg/g), LNC (leaf nitrogen content, %), LPC (leaf phosphorus content, %), and NP ratio (the ratio of leaf nitrogen and phosphorus content). All traits were measured following the protocols for standardized measurements of plant functional traits (Pérez‐Harguindeguy et al. 2013). Specifically, during late July–early August in 2021–2023, at the same time of frost tolerance sampling, we determined plant height as the maximum shoot height of each species in the field. Then we collected a total of five to ten fully developed leaves (including the petioles for eudicots) from five adult individuals of each species, depending on species abundance and leaf size, and combined for trait measurements. After transportation to the laboratory, leaves were carefully washed, water‐saturated fresh mass was weighed, and scanned using an EPSON V19 scanner (EPSON V19, Tokyo, Japan). The scanned images were analyzed for leaf area and then dried at 60°C for 48 h to a constant weight and weighed to calculate SLA and LDMC. Finally, the leaf samples were ground, LNC and LPC were measured by an elemental analyzer (Vario EL III; Elementar Inc., Hanau, Germany). NP ratio was calculated as the ratio of LNC and LPC. Five individuals of each species were pooled to obtain one value per species for each trait.

### 2.4 Statistical Analysis

#### 2.4.1 Data Processing

The lethal temperature at which 50% of the individuals across the temperature treatments experienced frost damage (LT_50_) was calculated from the following logistic regression model (Lim et al. 1998):

(2) $$y=\frac{k}{1+{\mathrm{ae}}^{-\mathrm{bx}}}$$

where *y* represents the relative conductivity under different temperature treatments, *k* represents the saturation value of the conductivity (the maximum value is 100), *x* indicates the freezing treatment temperature, *a* and *b* represent the regression coefficients. We linearized the equation to find the values of *a* and *b*, and LT_50_ that were obtained as follows:

(3) $$LT_{50}=\frac{\ln a}{b}$$

We repeated the experiment over three years (2021–2023), to ensure that the findings were generalizable and not specific to the conditions observed in one year. In 2021, LT_50_ and six functional traits of 13 species (*Aster diplostephioides, Aster hispidus, Carex moorcroftii, Gentiana parvula, Lagotis brachystachya, Phlomoides rotata, Lancea tibetica, Leontopodium pusillum, Oxytropis proboscidea, Dasiphora dryadanthoides, Saussurea andryaloides, Thalictrum alpinum, Veronica ciliata*) were measured; all seven traits of 22 target species were measured in 2022 and 2023. Five replicates (five individual plant samples) of seven traits were obtained for each species in each year. We used the species level data in the following analysis by averaging five replicate measurements in three years. All trait values were ln‐transformed to meet the assumptions of normality test. Since LT_50_ values are negative, we added 25 (a number lower than the lowest LT_50_ value of all species) for LT_50_ value of each species before ln‐transforming.

#### 2.4.2 Data Analysis

Variation in LT_50_ among twenty‐two target species was tested by means of a one‐way ANOVA with species as factor. This was done using species‐level data with five replicate measurements by averaging the value of three years.

To explore how frost tolerance and six functional traits in this study covary, we analyzed their relationship in the multivariate trait space and performed a principal component analysis (PCA) with ln‐transformed species‐wise mean values for all parameters. To find the best combination of traits that explain frost tolerance, we further performed multiple regressions, with ln LT_50_ as the response variable and trait values (ln height, ln SLA, ln LDMC, ln LNC, ln LPC, and ln NP ratio) as explanatory variables. Due to the multicollinearity of the NP ratio with LNC and LPC, two separate models were applied. Explanatory variables were ln height, ln SLA, ln LDMC, ln LNC, and ln LPC in the first model and ln height, ln SLA, ln LDMC, and ln NP ratio in the second model. For both models, full models were simplified via backward selection of the least significant variable until the final minimal adequate model (Akaike information criterion (AIC)) was found (Crawley 2012). The variance inflation factor (VIF) value of each variable was checked in multiple regressions to evaluate the collinearity, and no VIF was greater than 2. All of these analyses were done using species‐level data by averaging trait values of five replicates over three years.

Due to the large difference in LT_50_ of the species *Oxytropis proboscidea* relative to all other species, we conducted all the above analyses again using data without *O. proboscidea*, to examine the leveraging effect of *O. proboscidea*. These analyses were also done using species‐level data by averaging trait values of five replicates in three years and ln‐transformed.

For the analysis of one‐way ANOVA, multiple regressions, and the linear models, “lm” function was used. The “cor” function in the package “stats” was used for pairwise correlations and graphically displayed with “ggcorrplot2” (Cai et al. 2022). The PCA was computed using “prcomp” function and graphically displayed with “ggord” (Marcus 2024). Data were analyzed using R (R Core Team 2023). Figures were graphically displayed using ggplot2 (Wickham 2016).

## 3 Results

### 3.1 Frost‐Tolerance of Twenty‐Two Alpine Species

**FIGURE 1:**
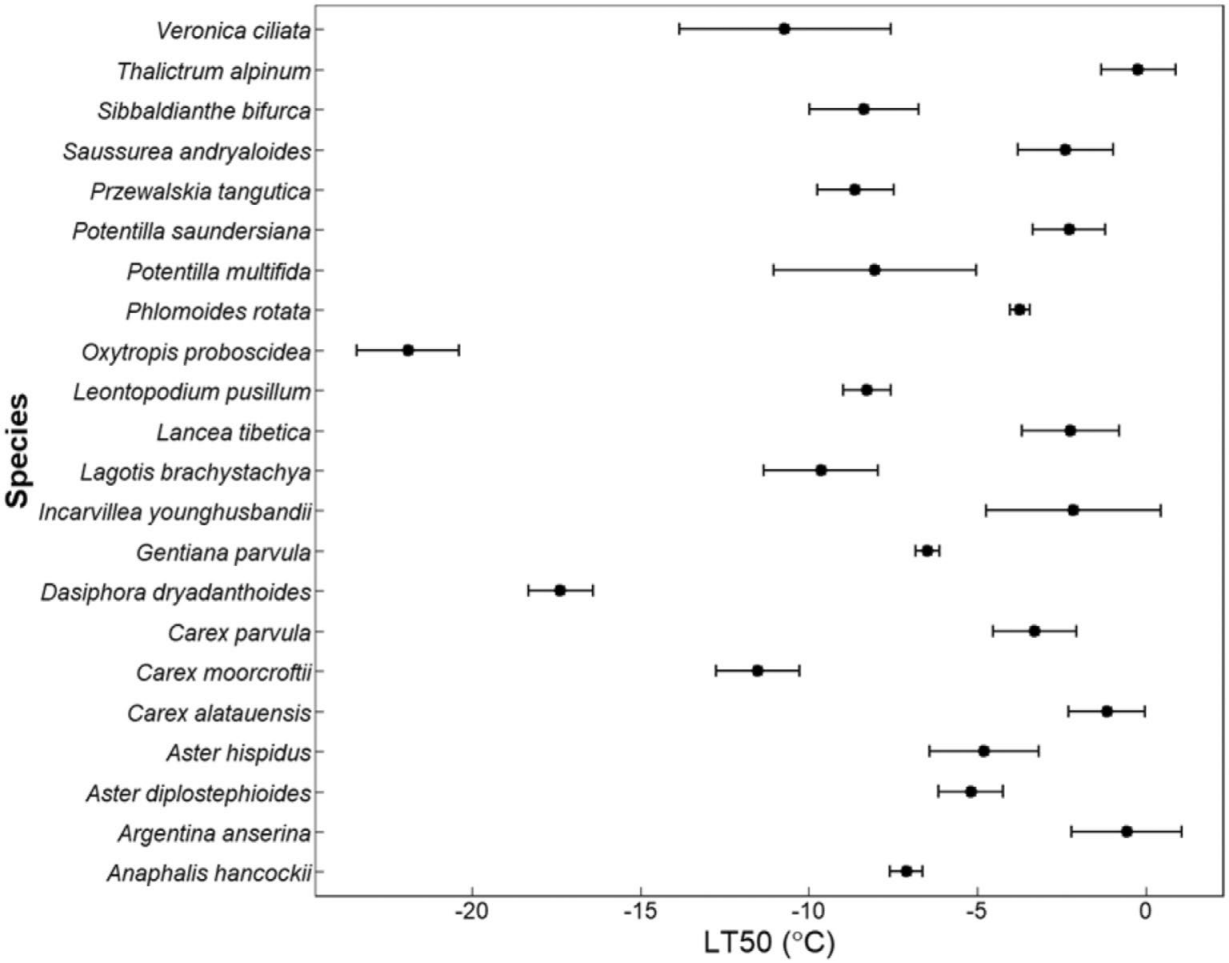
The frost tolerance (LT_50_ ± standard error) values of the twenty‐two selected species. 15 samples were used to calculate the mean and standard error of each species with 5 replicates in each year (2021–2023).

There were highly significant differences in frost tolerance (LT_50_) among the selected plants (Figure 1; Df = 21, *F* = 41.55; *p* < 0.0001), ranging from −21.89°C in *Oxytropis proboscidea* to −0.22°C in *Thalictrum alpinum* (lower LT_50_ value indicates higher frost‐tolerance). LT_50_ value of the only shrub in this study, *Dasiphora dryadanthoides* (−17.36°C), was higher than *O. proboscidea* and lower than all other species, while LT_50_ value of the only annual species, *Gentiana parvula* (−6.47°C) was lower than many perennial species (Figure 1). After removing *O. proboscidea* (having a much smaller LT_50_ value), differences in LT_50_ between species remained significant (Figure S3; Df = 20, *F* = 26.41; *p* < 0.0001).

### 3.2 Association of Frost‐Tolerance With Functional Traits

**FIGURE 2:**
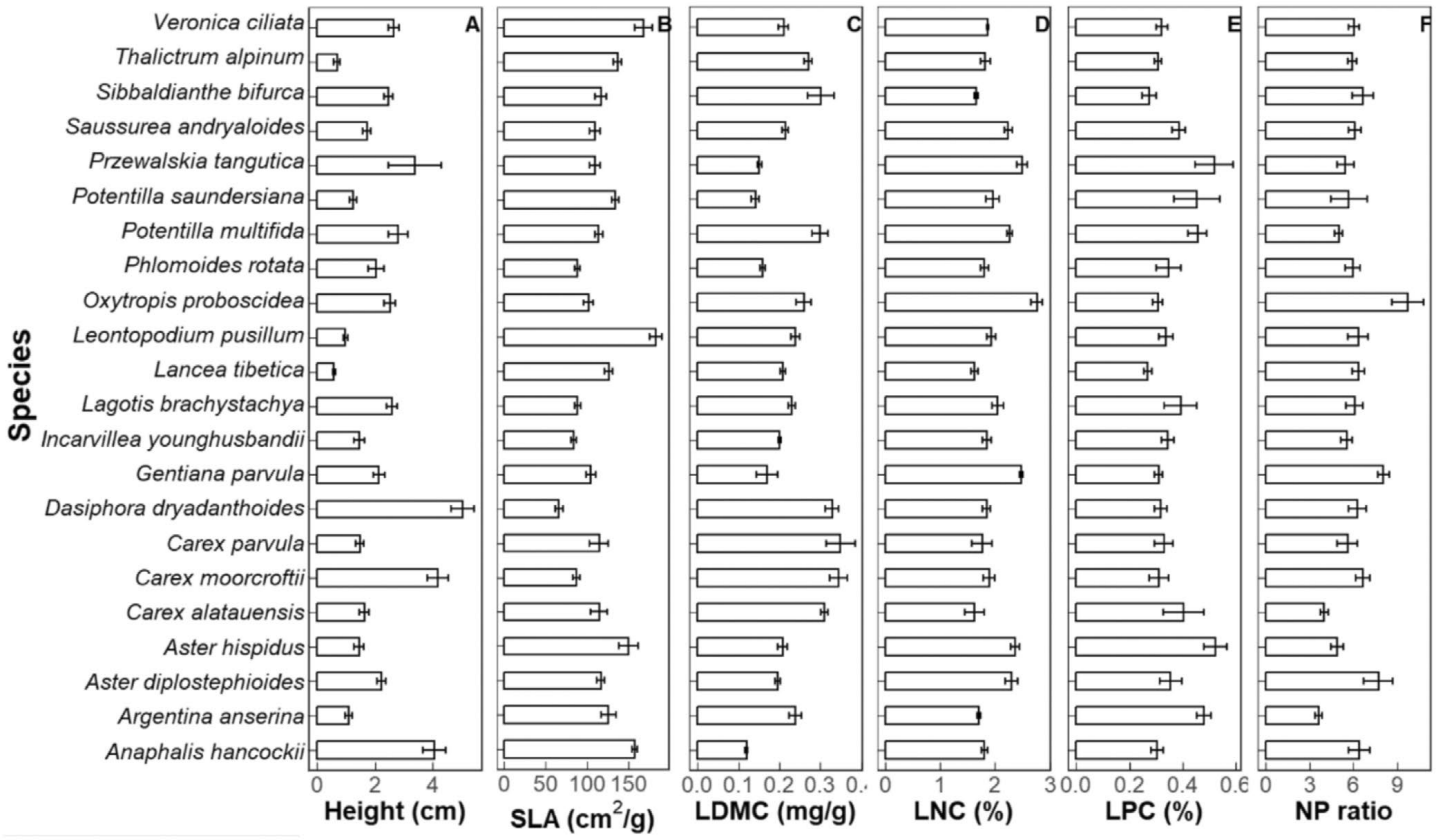
The values of functional traits in this study (mean ± standard error) of the twenty‐two species. 15 samples were used to calculate the mean and standard error of each species with 5 replicates in each year (2021–2023).

**FIGURE 3:**
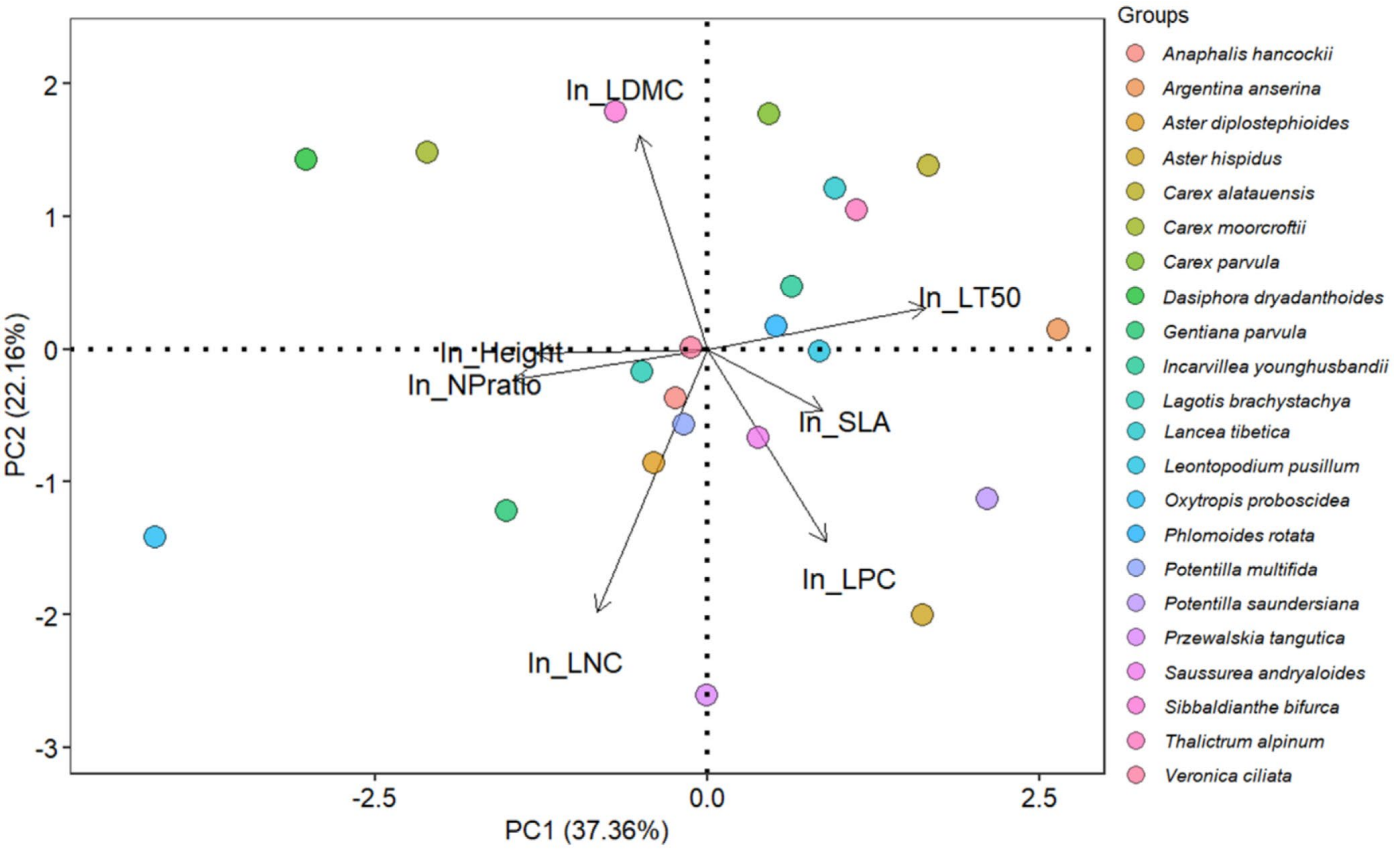
Principal component analysis of LT_50_ and six functional traits (height, SLA, LDMC, LNC, LPC, NP ratio). All trait values were ln‐transformed. Each point represents a species (*N* = 15).

PCA results showed that over 50% of all trait variation in this study was explained by the first two axes (Figure 3 and Table S3). The first axis showed a negative correlation between LT_50_ on one side and plant height and NP ratio on the other, whereas the second axis was mainly associated with growth traits, that is, LDMC, LNC and LPC (Figure 3 and Table S3). Tall species, namely *Carex moorcroftii* and *Dasiphora dryadanthoides*, and species with high LNC and NP ratio species *O. proboscidea* were more frost‐tolerant, while short species, *Argentina anserina* and *P. saundersiana* had larger LT_50_ value (Figures 2 and 3). The PCA results without *O. proboscidea* were similar to the above results, with LT_50_, plant height, NP ratio and SLA on the first axis, and LDMC, LNC and LPC on the second axis (Figure S4).

In the multiple regression of LT_50_ with plant height, SLA, LDMC, LNC, and LPC as predictors, height, LNC, and LPC had significant effects on LT_50_ (Table 1, model 1: AIC = 21.94; height: *p* = 0.006; LNC: *p* = 0.03; LPC: *p* = 0.04). As for the multiple regression of LT_50_ with plant height, SLA, LDMC, and NP ratio as predictors, plant height and NP ratio had significant effects on LT_50_ (Table 1, model 2: AIC = 22.39; height: *p* = 0.007; NP ratio: *p* = 0.009). In both cases, plant height was the most correlated trait to variation in LT_50_ (Table 1 and Figure 3). The results of the analysis without *O. proboscidea* were different, as plant height had an even stronger effect on LT_50_, while the effects of other traits were no longer significant (Table S2; model 3: AIC = −5.53; height: *p* < 0.0001; model 4: AIC = −3.60; height: *p* < 0.0001; Figure S5).

**TABLE 1:** Multiple regression analysis shows the association of LT_50_ with functional traits.

Model 1				Model 2			
Trait	Df	*F*	*p*	Trait	Df	*F*	*p*
ln Height	1	10.11	0.006**	ln Height	1	9.61	0.007**
ln SLA	1	0.35	0.56	ln SLA	1	0.33	0.57
ln LDMC	1	1.77	0.20	ln LDMC	1	1.68	0.21
ln LNC	1	5.96	0.03*	ln NP ratio	1	8.60	0.009*
ln LPC	1	4.96	0.04*	/	/	/	/

*Note:* All trait values were ln‐transformed. Due to multicollinearity of NP ratio with LNC and LPC, two separate models were applied. Model 1 includes height, SLA, LDMC, LNC, and LPC as explanatory variables, model 2 includes height, SLA, LDMC, and NP ratio. Degree of freedom (Df), *F* value, and *p* value were shown in the table. Bold values indicate significant effects at 0.05 level, ** and * indicate significance at 0.01 and 0.05 level, respectively.

**FIGURE 4:**
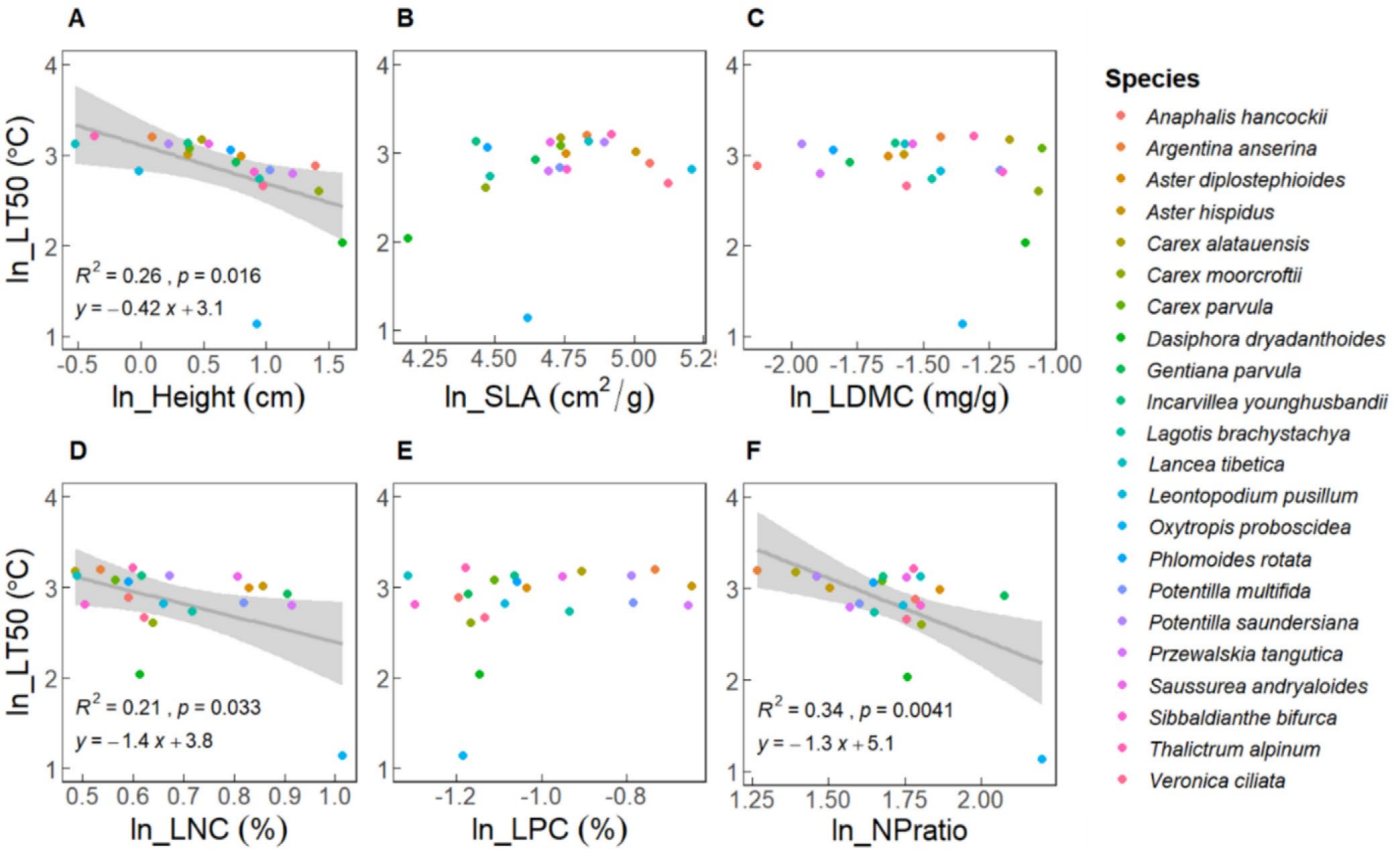
The pairwise relationship between LT_50_ and six functional traits (height, SLA, LDMC, LNC, LPC, NP ratio). All trait values were ln‐transformed. Each point represents a species (*N* = 15), the gray lines represent linear model fits, the shaded area is the 95% confidence interval band. Significant linear relationships were shown with equation, *R* ^2^, and *p* value.

We found that *O. proboscidea* had a leveraging effect on the relationship of LT_50_ with LNC and NP ratio. *O. proboscidea* had a much smaller LT_50_ value, significantly differing from all other species in this study (Figure 1). LNC and NP ratio were negatively associated with LT_50_, however, they were no longer significantly related to LT_50_ when excluding *O. proboscidea* from the species pool in statistical models (Table 1 and Figure 4; Table S2 and Figure S5). These results indicated that LNC and NP ratio were related to LT_50_ only because of high LNC in *O. proboscidea* . Moreover, it implied that the close relationship between LT_50_ and plant height was robust; however, LNC and NP ratio, rather than height, determined the frost tolerance of *O. proboscidea* .

## 4 Discussion

The frost‐tolerance of twenty‐two alpine plant species from the Tibetan Plateau based on LT_50_ showed significant interspecific variation. Plant height, out of six functional traits, was the main trait associated with frost‐tolerance. However, opposite to our initial expectation, we found taller plant species to be more frost‐tolerant. Our study indicates that plants of different heights co‐occurring in the same community may adopt contrasting frost‐resistance strategies to cope with low temperature in alpine ecosystems, as short species are more likely to develop a frost‐avoidance strategy, and taller species a frost‐tolerance strategy.

### 4.1 Frost‐Tolerance Is Closely Related to Plant Height

In line with our first hypothesis, frost tolerance of the studied species showed a striking interspecific difference, as LT_50_ ranged from −21.89°C to −0.22°C. This result shows that the variation within a single high elevation habitat (4450 to 4550 m) is comparable with the average difference observed along a 1000 m elevation gradient (Bucher et al. 2019; Bucher and Rosbakh 2021; Gast et al. 2020). Our finding is consistent with Blonder et al. (2020), that the filtering effect of low temperatures does not preclude the diversity of strategies for co‐occurring species within a single community in high elevation alpine areas.

We demonstrated that frost‐tolerance was negatively related to plant height, indicating that taller plants were more frost‐tolerant. This finding was contrary to our second hypothesis and a previous study along an elevation gradient in the Alps, that alpine species with higher canopy height had weaker frost‐tolerance (Bucher and Rosbakh 2021). Our result was also inconsistent with Sklenář et al. (2010), who found that short species tolerate freezing and taller species avoid them. It was based on a thermal gradient in the air‐soil profile, where colder air masses accumulated near soil surfaces during clear sky nights, exposing prostrate species to more extreme thermal conditions than those with canopies well above the ground (Sierra‐Almeida et al. 2010; Squeo et al. 1991). Our results are highly consistent with those of Liancourt et al. (2020), that plant height was largely related to the extent of temperature decoupling in alpine plants in the Himalaya region, as a consequence of heat transfer influenced by radiation, convection, and transpiration (Dong et al. 2017; Michaletz et al. 2015, 2016). In other words, short‐statured plants are aerodynamically decoupled from the cold air temperature (Liancourt et al. 2020; Körner 2021). Moreover, these short plants also share a common syndrome of alpine dwarfism, such as dense canopy, cushion or carpet forming growth form and small leaves that can all contribute to warmer temperature than ambient air (Dolezal et al. 2019). Finally, shorter plants are more likely to benefit from the thermal heterogeneity of the alpine terrain (Scherrer and Körner 2011). Taken together, we showed that short plants experience much warmer temperature than taller plants, thus require less to be frost‐tolerant, and are more likely to develop a frost avoidance strategy.

This finding therefore implies that plant species in the same alpine community may adopt different strategies to cope with frost stress (Blonder et al. 2020), either frost‐tolerance or frost‐avoidance. Low temperature stress mainly damages plants through the formation of ice crystals on plant tissues when the temperature drops to or below freezing point (Sakai and Larcher 1987; Larcher 2003). Supercooling enables plant cells to avoid frost and retain a liquid state at temperatures below freezing point (Sakai and Larcher 1987; Wisniewski et al. 2009, 2014). However, when the temperature drops to a certain threshold, supercooling may not necessarily protect plants from freezing damage (Larcher and Bauer 1981; Larcher et al. 2010). It is then necessary to switch from a frost‐avoidance to a frost‐tolerance strategy, that is, form ice crystals in extracellular spaces, which prevents ice occurrence and subsequent damage in the living cells (Körner 2021; Sklenář 2017). Experimentally examining and comparing supercooling point and frost‐tolerance (LT_50_) can determine which strategy each species is taking (Sakai and Larcher 1987; Sklenář 2017). Generally in cold environments, a more negative LT_50_ indicates frost‐tolerance as a mechanism of resistance, while a less negative LT_50_ may indicate frost‐avoidance (Sklenář 2017). Our result is consistent with the recent observations of the diversity of strategies found in other extreme environments like drylands (Gross et al. 2024), as both drought tolerance and avoidance strategies are present in a single community. Thus multiple ways to deal with local stress likely benefit community structure and ecosystem functioning (Kramp et al. 2022; Markesteijn et al. 2011; Mitchell et al. 2008). Further experiments are needed to measure the supercooling point of each species, compare it to LT_50_, thereby confirming the tolerance or avoidance strategy of alpine species on the Tibetan Plateau and elsewhere.

### 4.2 No Relationship Was Found Between Frost‐Tolerance and Resource Economy Related Traits

Our third hypothesis was also rejected. We did not observe a trade‐off between frost‐tolerance and exploitive strategy related trait SLA. We also did not observe a negative relationship between LT_50_ and LDMC, a conservative strategy related trait. These observations indicate that the frost‐tolerance of co‐occurring alpine plants were determined by plant size related traits (i.e., height), not by resource economy related traits (i.e., SLA and LDMC). Thus, the relationship between frost‐tolerance and resource economy traits observed within a community was different from the relationship observed along an elevation gradient. This pattern is consistent with a previous study revealing that, due to adaptive variation or compositional shift, a trait‐environment relationship is not always universal (Meng et al. 2015). We suggest that caution is needed when inferring difference in plant strategies, like tolerance within communities, from trait variation along gradients. Differences among spatial scales should be considered in trait‐ environment relationship studies.

We found that *O. proboscidea* had a leveraging effect on the relationship of LT_50_ with LNC and NP ratio. LNC is generally closely related to Rubisco content and photosynthetic capacity (Chai et al. 2024; Luo et al. 2021), thus fast‐growing plants with high LNC are generally considered to be less frost‐tolerant (Bucher et al. 2018; Bucher and Rosbakh 2021). However, we found the opposite pattern, as *O. proboscidea* had a high LNC and high NP ratio, which was driven by LNC. Previous studies also indicate that LNC is an important component in cryoprotectants (proline and antifreeze proteins) (Hoshino et al. 1999; Liu et al. 2021; Meza‐Basso et al. 1986). This mechanism may be why *O. proboscidea* was more frost‐tolerant than all the other species observed in this study. Additional research on *O. proboscidea* is required to identify the driver of its frost‐tolerance and explore the underlying mechanisms.

## 5 Conclusion and Implications

Overall, frost tolerance of twenty‐two species co‐occurring within a single community showed a relatively high interspecific variation that was closely related to plant height, out of six functional traits. No trade‐off was found between frost tolerance and plant growth, as LT_50_ was not correlated with SLA and LDMC. A leveraging effect of *O. proboscidea* was found on the relationship between LT_50_ and LNC and NP ratio. Our study highlights that contrasting morphological and physiological adaptations may explain the mechanisms of species co‐existence in an alpine meadow community. Unlike the taller plants from the community, short‐stature and cushion species are inclined to employ avoidance strategies when facing mild frost and do not have to rely on physiological tolerance to frost. This is important when understanding species co‐existence in an alpine community, as multiple strategies can be equally efficient under harsh conditions and ultimately have positive impacts on community structure and ecosystem functioning.

This study also has limitations. Because we only measured frost tolerance of plant species and did not simultaneously measure frost avoidance, the differences in frost resistance strategies among species and their mechanisms underlying species coexistence in the community were discussed and inferred based on previous studies. Therefore, future research still needs to simultaneously measure both frost avoidance and frost tolerance indices for each species to validate the findings of this study, thereby more clearly elucidating the role of plant frost resistance strategies in structuring alpine meadow communities. Furthermore, future research also needs to investigate the relationship between the non‐structural carbohydrate content (including soluble sugars and starch), which is directly associated with plant stress resistance, and frost resistance strategies of alpine plants, thereby understanding the freezing resistance mechanisms of alpine plants from the perspective of plant physiological changes.

Our quantitative research on frost‐tolerance of twenty‐two plant species commonly found across the Tibetan Plateau is particularly valuable in plant physiological models related to distribution range, such as process‐based distribution models. Frost‐tolerance of a given species represents its low‐temperature range limits and mostly drives its natural distribution range (Araújo et al. 2013). Therefore, field measurements from remote areas make model fitting and prediction more accurate at the macroscale level. Given the increasing probability of modification in the frequency of frost events with climate change, these results are of great significance for accurately assessing and forecasting the effect of climate on species distributions.

## Author Contributions

**Ji Suonan:** conceptualization (equal), data curation (equal), formal analysis (equal), funding acquisition (equal), investigation (equal), methodology (equal), project administration (equal), resources (equal), software (equal), supervision (equal), validation (equal), visualization (equal), writing – original draft (equal), writing – review and editing (equal). **Xuwei Lu:** data curation (equal), formal analysis (equal), writing – original draft (equal). **Richard Michalet:** conceptualization (equal), writing – original draft (equal). **Pierre Liancourt:** conceptualization (equal), writing – original draft (equal). **Aimée T. Classen:** writing – original draft (equal). **Wenying Wang:** project administration (equal), resources (equal), writing – original draft (equal). **Huichun Xie:** project administration (equal), resources (equal), writing – original draft (equal).

## Funding

This work was supported by the Ministry of Education, the Ministry of Science and Technology, and the State Administration of Foreign Experts Affairs of China (D23029), National Natural Science Foundation of China (31800407, 32160285, and 32560295).

## Conflicts of Interest

The authors declare no conflicts of interest.

## Supporting information


**Data S1:** LT50ANDTRAITS.

**Figure S1:** Extreme cooling events during the growing season of 2011–2019 in Nagqu.
**Figure S2:** The seven target temperatures set in this study and the actual temperature achieved (The orange curves are the actual temperature recorded in the chamber, and the black curves are the preset temperature).
**Figure S3:** The frost tolerance (LT_50_ ± standard error) values of the twenty‐one species (without *Oxytropis proboscidea*). 15 samples were used to calculate the mean and standard error of each species with 5 replicates in each year (2021–2023).
**Figure S4:** Principal component analysis of LT50 and six functional traits (height, SLA, LDMC, LNC, LPC, NP ratio) of twenty‐one species (without *O. proboscidea* ). All trait values were ln‐transformed. Each point represents a species (*N* = 15).
**Figure S5:** The pairwise relationship between LT_50_ and six functional traits (height, SLA, LDMC, LNC, LPC, NP ratio) of twenty‐one species (without *O. proboscidea* ). All trait values were ln‐transformed. Each point represents a species (*N* = 15), the gray lines represent linear model fits, the shaded area is the 95% confidence interval band. Significant linear relationships were shown with equation, *R* ^2^, and *p* value.
**Table S1:** The twenty‐two selected species in this study with their family and genus.
**Table S2:** Multiple regression analysis shows the association of LT_50_ with functional traits of twenty‐one species (without *O. proboscidea* ). All trait values were ln‐transformed. Due to multicollinearity of N:P with LNC and LPC, two separate models were applied. Model 3 includes height, SLA, LDMC, LNC, and LPC as explanatory variables, model 4 includes height, SLA, LDMC, and NP ratio. Degree of freedom (Df), *F* value, and *p* value were shown in the table. Bold values indicate significant effects at 0.05 level, *** indicates significance at 0.001 level.
**Table S3:** The loadings of each variable on each principal component.

## Data Availability

The data that support the findings of this study are provided as Supporting Information.
