# Supplementary material for: Frost Tolerance Increases With Plant Height Among Co‐Occurring Alpine Species in the Central Tibetan Plateau
Source: Ecol Evol. 2026 Apr 29;16(5):e73512. doi: 10.1002/ece3.73512 (PMC13126090; doi:10.1002/ece3.73512)
Supplement: Supplementary file 2 — Figure S1: Extreme cooling events during the growing season of 2011–2019 in Nagqu. Figure S2: The seven target temperatures set in this study and the actual temperature achieved (The orange curves are the actual temperature recorded in the chamber, and the black curves are the preset temperature). Figure S3: The frost tolerance (LT50 ± standard error) values of the twenty‐one species (without Oxytropis proboscidea). 15 samples were used to calculate the mean and standard error of each species with 5 replicates in each year (2021–2023). Figure S4: Principal component analysis of LT50 and six functional traits (height, SLA, LDMC, LNC, LPC, NP ratio) of twenty‐one species (without O. proboscidea ). All trait values were ln‐transformed. Each point represents a species (N = 15). Figure S5: The pairwise relationship between LT50 and six functional traits (height, SLA, LDMC, LNC, LPC, NP ratio) of twenty‐one species (without O. proboscidea ). All trait values were ln‐transformed. Each point represents a species (N = 15), the gray lines represent linear model fits, the shaded area is the 95% confidence interval band. Significant linear relationships were shown with equation, R 2, and p value. Table S1: The twenty‐two selected species in this study with their family and genus. Table S2: Multiple regression analysis shows the association of LT50 with functional traits of twenty‐one species (without O. proboscidea ). All trait values were ln‐transformed. Due to multicollinearity of N:P with LNC and LPC, two separate models were applied. Model 3 includes height, SLA, LDMC, LNC, and LPC as explanatory variables, model 4 includes height, SLA, LDMC, and NP ratio. Degree of freedom (Df), F value, and p value were shown in the table. Bold values indicate significant effects at 0.05 level, *** indicates significance at 0.001 level. Table S3: The loadings of each variable on each principal component. [file ECE3-16-e73512-s002.docx]

Supporting information

Figure S1. Extreme cooling events during the growing season of 2011–2019 in Nagqu.


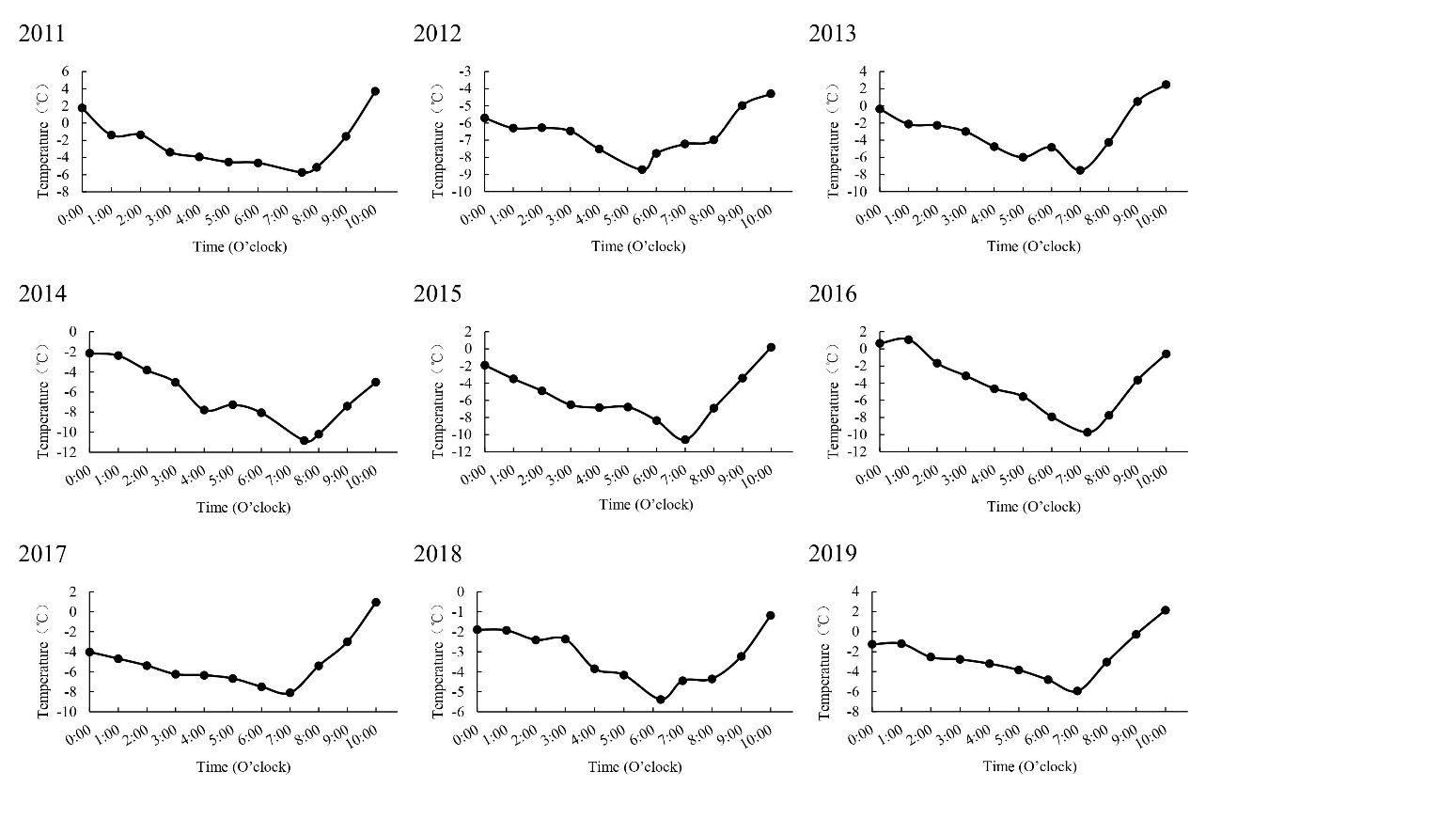


Figure S2. The seven target temperatures set in this study and the actual temperature achieved (The orange curves are the actual temperature recorded in the chamber, and the black curves are the preset temperature)


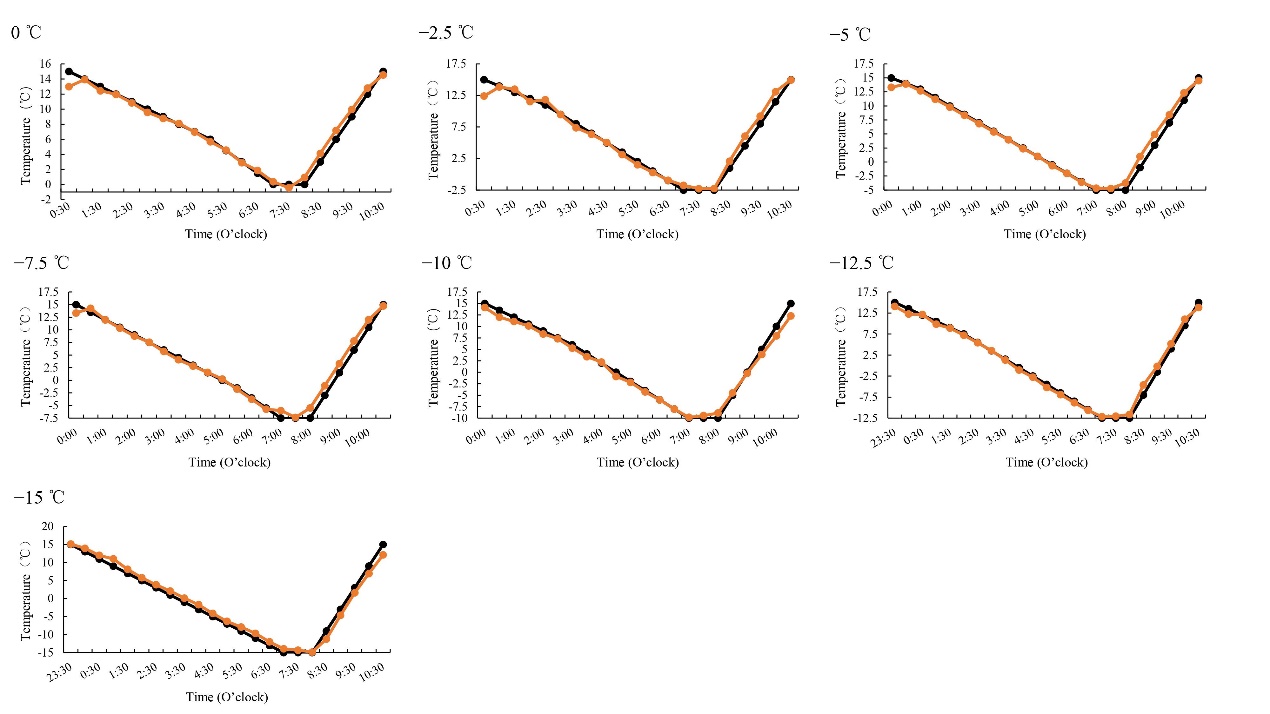


Figure S3. The frost tolerance (LT_50_ ± standard error) values of the twenty-one species (without *Oxytropis proboscidea*). 15 samples were used to calculate the mean and standard error of each species with 5 replicates in each year (2021-2023).


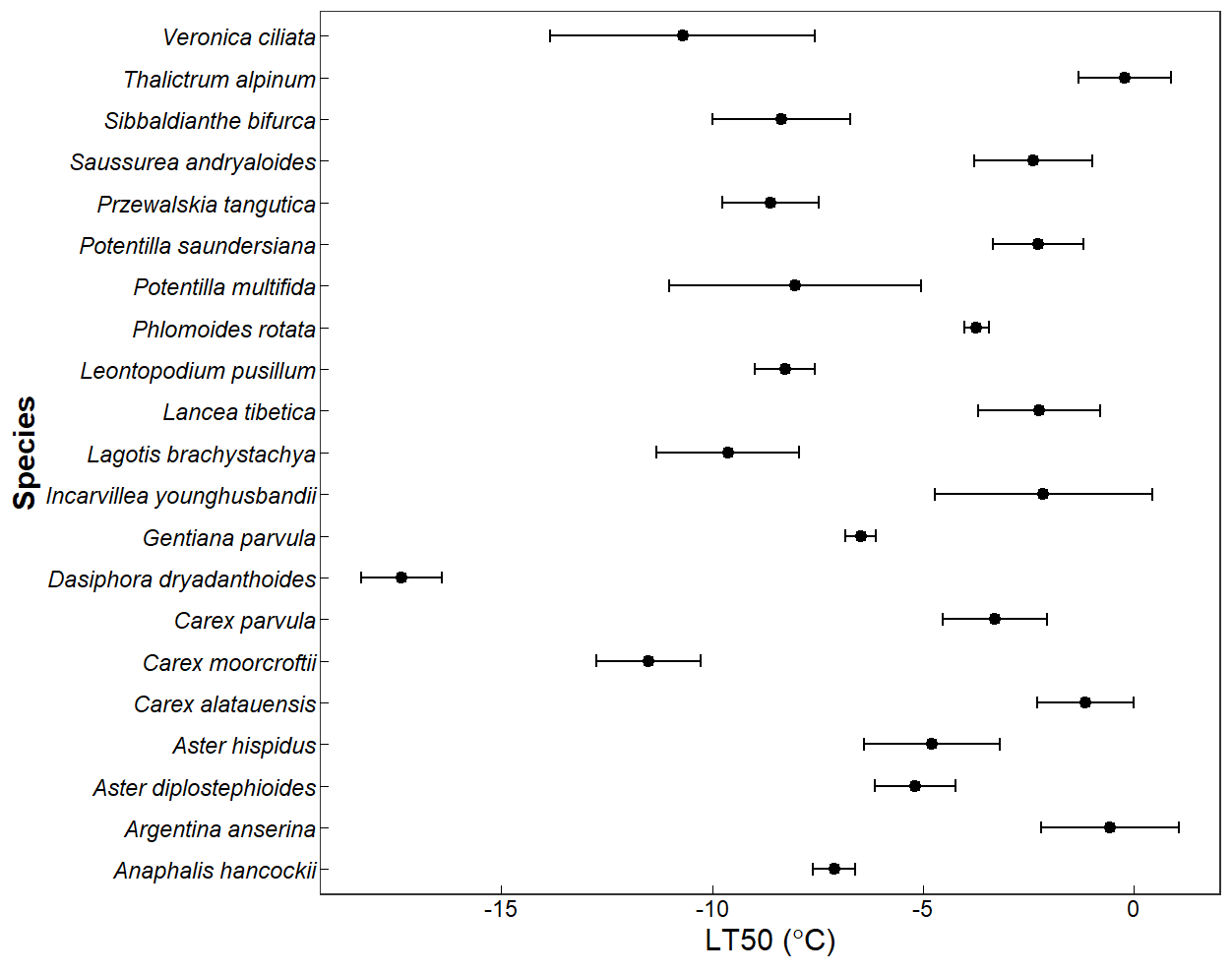


Figure S4. Principal component analysis of LT50 and six functional traits (height, SLA, LDMC, LNC, LPC, NP ratio) of twenty-one species (without *O. proboscidea*). All trait values were ln-transformed. Each point represents a species (N = 15).


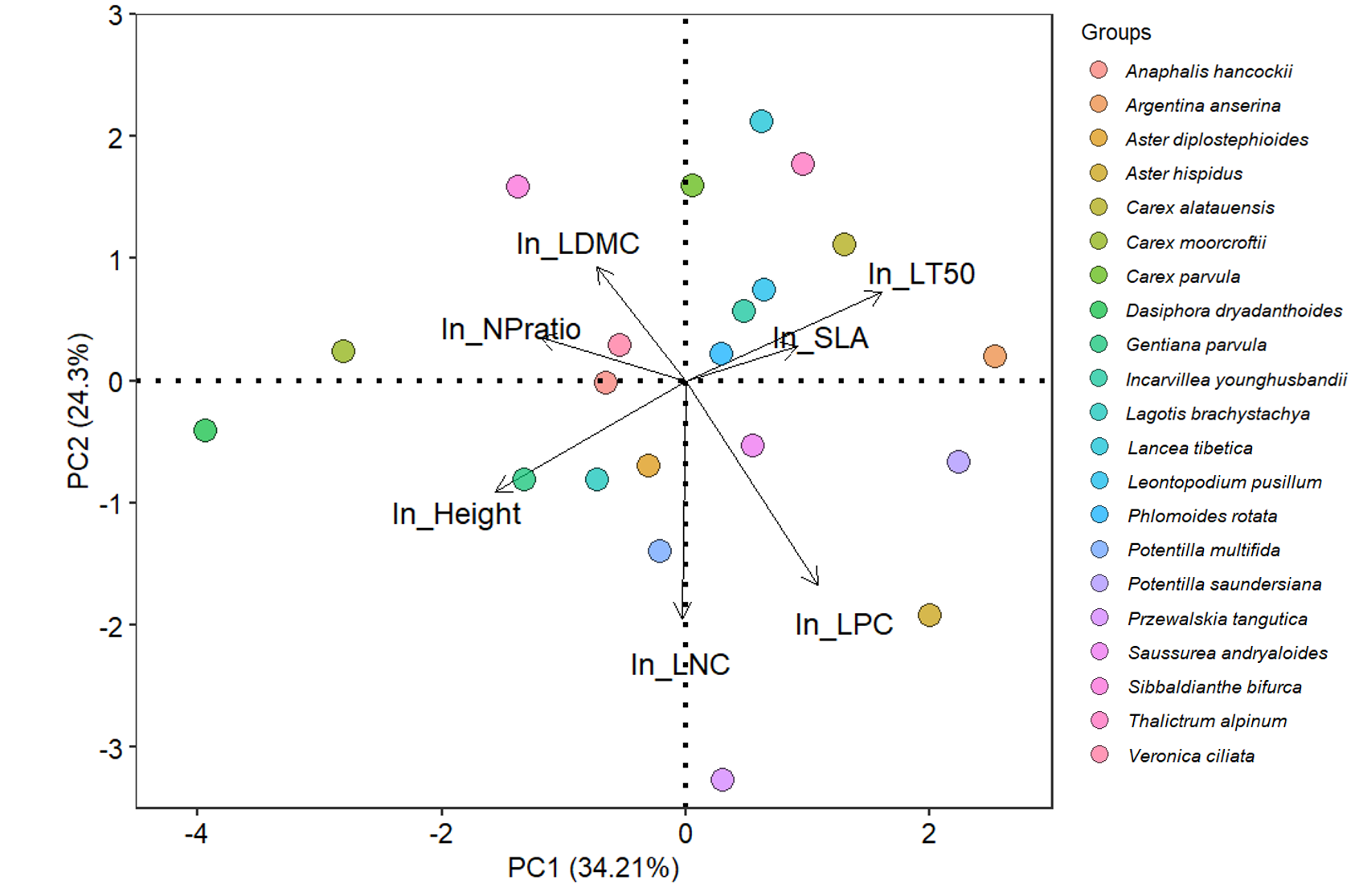


Figure S5. The pairwise relationship between LT_50_ and six functional traits (height, SLA, LDMC, LNC, LPC, NP ratio) of twenty-one species (without *O. proboscidea*). All trait values were ln-transformed. Each point represents a species (N = 15), the grey lines represent linear model fits, the shaded area is the 95% confidence interval band. Significant linear relationships were shown with equation, R^2^, and p value.


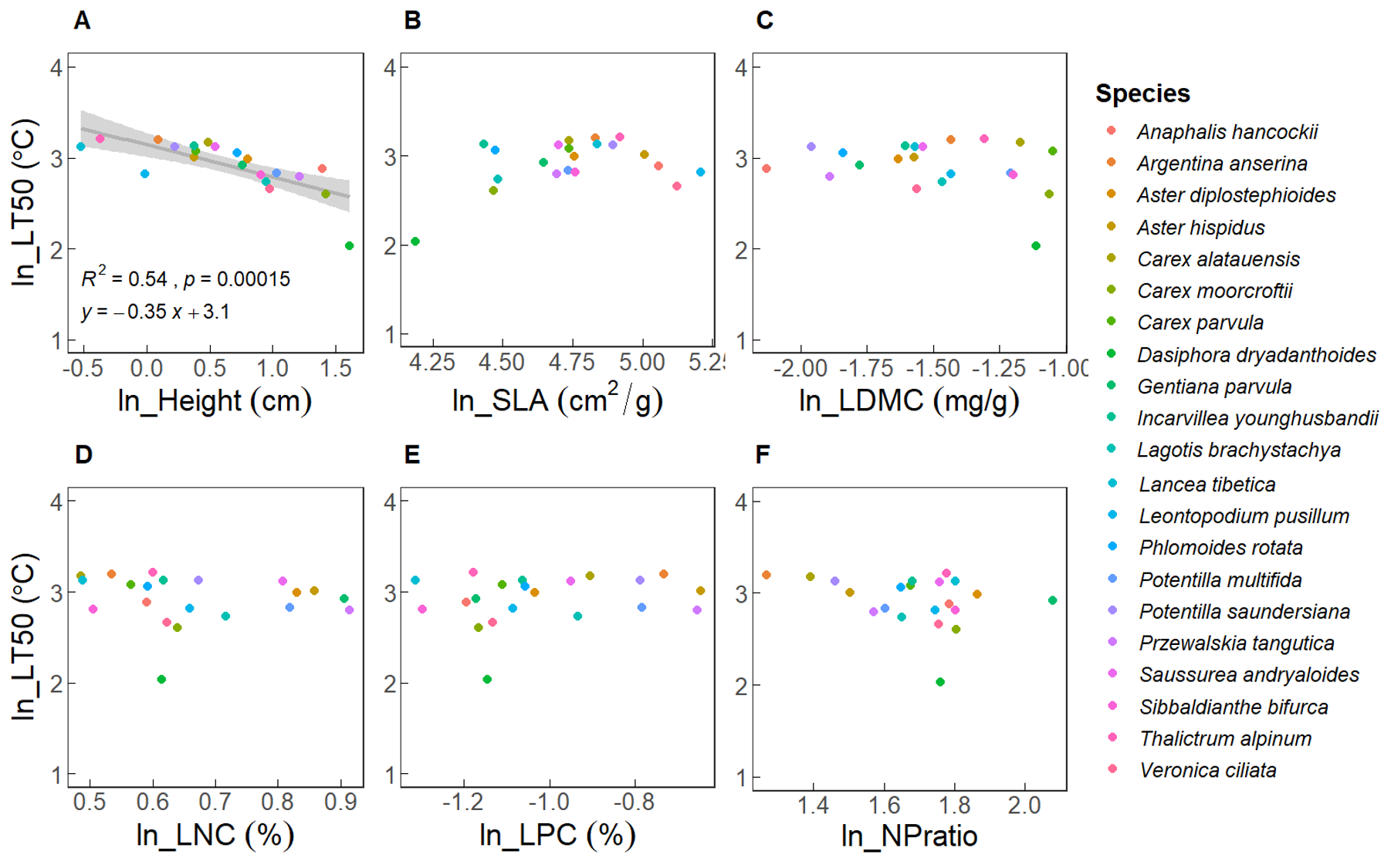


Table S1. The twenty-two selected species in this study with their family and genus.

| Number | Species | Family | Genus |
| --- | --- | --- | --- |
| 1 | *Anaphalis hancockii* | Asteraceae | Anaphalis |
| 2 | *Argentina anserina* | Rosaceae | Argentina |
| 3 | *Aster diplostephioides* | Asteraceae | Aster |
| 4 | *Aster hispidus* | Asteraceae | Aster |
| 5 | *Carex alatauensis* | Cyperaceae | Carex |
| 6 | *Carex moorcroftii* | Cyperaceae | Carex |
| 7 | *Carex parvula* | Cyperaceae | Carex |
| 8 | *Dasiphora dryadanthoides* | Rosaceae | Dasiphora |
| 9 | *Gentiana parvula* | Gentianaceae | Gentiana |
| 10 | *Incarvillea younghusbandii* | Bignoniaceae | Incarvillea |
| 11 | *Lagotis brachystachya* | Plantaginaceae | Lagotis |
| 12 | *Lancea tibetica* | Mazaceae | Lancea |
| 13 | *Leontopodium pusillum* | Asteraceae | Leontopodium |
| 14 | *Oxytropis proboscidea* | Fabaceae | Oxytropis |
| 15 | *Phlomoides rotata* | Lamiaceae | Phlomoides |
| 16 | *Potentilla multifida* | Rosaceae | Potentilla |
| 17 | *Potentilla saundersiana* | Rosaceae | Potentilla |
| 18 | *Przewalskia tangutica* | Solanaceae | Przewalskia |
| 19 | *Saussurea andryaloides* | Asteraceae | Saussurea |
| 20 | *Sibbaldianthe bifurca* | Rosaceae | Sibbaldianthe |
| 21 | *Thalictrum alpinum* | Ranunculaceae | Thalictrum |
| 22 | *Veronica ciliata* | Plantaginaceae | Veronica |

Table S2. Multiple regression analysis shows the association of LT_50_ with functional traits of twenty-one species (without *O. proboscidea*). All trait values were ln-transformed. Due to multicollinearity of N:P with LNC and LPC, two separate models were applied. Model 3 includes height, SLA, LDMC, LNC, and LPC as explanatory variables, model 4 includes height, SLA, LDMC, and NP ratio. Degree of freedom (Df), F value, and P value were shown in the table. Bold values indicate significant effects at 0.05 level, *** indicates significance at 0.001 level.

| Model 1 | | | | Model 2 | | | |
| --- | --- | --- | --- | --- | --- | --- | --- |
| Trait | Df | F | P | Trait | Df | F | P |
| ln Height | 1 | 22.57 | **<.001***** | ln Height | 1 | 24.00 | **<.001***** |
| ln SLA | 1 | 0.20 | 0.66 | ln SLA | 1 | 0.22 | 0.65 |
| ln LDMC | 1 | 2.69 | 0.12 | ln LDMC | 1 | 2.86 | 0.11 |
| ln LNC | 1 | 0.00 | 0.98 | ln NP ratio | 1 | 1.41 | 0.25 |
| ln LPC | 1 | 1.37 | 0.26 | / | / | / | / |

Table S3 The loadings of each variable on each principal component.

| Trait | PC1 | PC2 |
| --- | --- | --- |
| ln LT50 | -0.538 | -0.084 |
| ln Height | 0.396 | 0.271 |
| ln SLA | -0.321 | 0.016 |
| ln LDMC | 0.143 | -0.408 |
| ln LNC | 0.296 | 0.588 |
| ln LPC | -0.296 | 0.617 |
| ln NP ratio | 0.505 | -0.160 |
